# Atopic Dermatitis-Like Skin Lesions Reduced by Topical Application and Intraperitoneal Injection of Hirsutenone in NC/Nga Mice

**DOI:** 10.1155/2010/618517

**Published:** 2010-12-14

**Authors:** Mi Sook Jeong, Sun Eun Choi, Ji Young Kim, Jung Shim Kim, Eun Joo Kim, Kwan Hee Park, Do Ik Lee, Seong Soo Joo, Chung Soo Lee, Hyoweon Bang, Mi-Kyung Lee, Young Wook Choi, Kap-sok Li, Nam Ju Moon, Min Won Lee, Seong Jun Seo

**Affiliations:** ^1^Department of Dermatology, College of Medicine, Chung-Ang University, Seoul 156-756, Republic of Korea; ^2^College of Pharmacy, Chung-Ang University, Seoul 156-756, Republic of Korea; ^3^Division of Marine Molecular Biotechnology, Gangneung-Wonju National University, 120 Gangneung Daehangno, Gangneung, Gangwon 210-702, Republic of Korea

## Abstract

Atopic dermatitis (AD) is a common inflammatory skin disease. The increasing prevalence and severity of AD have prompted the developments of safer, more effective drugs. Although topical corticosteroids have been used as first line therapy for AD, their potential side effects limit their clinical applications. To investigate the effect of hirsutenone (HIR), a diarylheptanoid compound, on AD-like skin lesions and other factors related to immune response is the aim of this paper Th2-related cytokines (IL-4, IL-5, IL-13), eosinophil, IgE inflammatory factors (COX-2, iNOS) levels were reduced in blood, lymphocytes, and tissue after HIR treatment. These results suggest that HIR might be an effective treatment for AD.

## 1. Introduction

Atopic dermatitis (AD) is a chronic relapsing skin disorder with allergic inflammation. AD is one of the most common skin diseases in children with a family history of atopy and is frequently associated with elevated plasma levels of IgE antibodies against inhaled allergens [1, 2]. The histology of AD is characterized by epidermal alterations and a dermal inflammatory infiltrate containing eosinophils [1]. The causes of atopic dermatitis are not completely understood, but a complex inflammatory immune dysregulation and response to allergens are believed to be involved [2]. 

 The most promising antiatopic dermatitis drugs are compounds that are immune-suppressive. These topical corticosteroids are the primary choice for AD treatment, but their side effects, such as, perioral dermatitis and skin atrophy and striae in sensitive areas, are a major obstacle to their long-term application [3]. 

 Recently, we isolated diarylheptanoid compounds from the bark of *Alnus japonica* [4]. The bark of *Alnus japonica* is used in oriental traditional medicine to treat fever, hemorrhage, diarrhea, gastroenteric disorder, lymphatic disease, and cancers [5]. The diarylheptanoids, which are characteristic components of *Alnus* species, have been reported to have several biological activities. In this study, we investigated HIR, a diarylheptanoid, which has previously been shown to have inhibitory activity on cyclooxygenase-2 expression and anti-inflammatory effects [6–12]. Furthermore, HIR has been reported to prevent cytokine and chemokine-mediated immune cell function and inflammatory reaction and was found to be an attractive starting point for the development of a topical drug for T cell-based anti-atopic dermatitis due to its calcineurin inhibitory effects [13, 14].

AD is frequently associated with elevated plasma levels of IgE antibodies against many kinds of inhaled allergens [15, 16]. IgE-mediated mast cell activation leads to the release of various chemical mediators, which results in the infiltration of inflammatory cells, such as, eosinophils and lymphocytes, into skin lesions. Moreover, when promoted by IL-5, IL-4 is able to trigger IgE synthesis and IL-4-dependent IgE synthesis in B cell [17]. In patients with AD, decreased IFN-*γ* production is considered to be associated with IgE hypersynthesis and Th2 immune response [18]. 

 In the present study, we induced AD-like skin lesions in NC/Nga mice by repeatedly applying *Dermatophagoides farina *(House dust) containing cream. We then examined whether HIR has an immune modulating effect in this model and compared this with those of the established therapeutic agents dexamethasone (DEX) and hydrocortisone cream (HDC). The efficacies of these test agents were evaluated using clinical skin severity scores, cytokine (IL-4, IL-5, IL-13) expression in blood and spleen, and total plasma levels of IgE. In an attempt to identify the molecular mode of action of HIR, we also examined its effect on the expressions of COX-2 and iNOS in skin.

## 2. Materials and Methods

### 2.1. Phytochemical

#### 2.1.1. Extraction and Isolation of HIR

The bark of *A. japonica* was collected at Mt. Sudal, Seoul, Republic of Korea in June 2008, and a voucher specimen (AJB0806) was deposited at the herbarium, College of Pharmacy, Chung-Ang University. Bark (5.15 kg) was extracted for 72 h at room temperature with 80% aqueous acetone. After removing the acetone under vacuum, the aqueous solution was filtered through filter paper (Tokyo Roshi Kaisha Ltd, Japan), and the filtrate was concentrated and applied to a Sephadex LH-20 column (10–25 *μ*m, GE Healthcare Bio-Science AB, Uppsala, Sweden), and eluted with H_2_O containing increasing proportions of methanol to afford 4 fractions, A (44.31 g), B (173.94 g), C (2.12 g), and D (6.8 g). Repeated column chromatography of fraction B (173.94 g) on MCl-Gel CHP 20P (75–150 *μ*m, 5 × 80 cm, Mitsubishi Chemical Co., Tokyo) using an H_2_O: methanol gradient yielded oregonin (ORE) (39.99 g).

#### 2.1.2. Preparation of HIR by the Enzymatic Hydrolysis of ORE

The ORE (1 g, 1%, w/w) was diluted in distilled water (940 ml, 94%, w/w) and Pectinex AFP-L4 (polygalacturonase from *Aspergillus aculeatus* or *Aspergillus niger*) (Nobozymes Co. Ltd, Bagsvaerd, Denmark) (50 ml, 5%, w/w) was added [19, 20]. The mixture was then shaken aerobically at 150 rpm for 18 hours at room temperature, heated at 85°C for 5 min to inactivate the enzyme, and then centrifuged (3000 rpm) for 30 min and filtered. The filtrate was fractionated with ethyl acetate, and the ethyl acetate layer (0.63 g) was applied to a Sephadex LH-20 column and eluted with 60% MeOH to yield HIR (0.252 g).

#### 2.1.3. ORE (1.7-bis-(3,4-Dihydroxy-Phenyl)-Heptane-3-on-5-O-*β*-D-Xylopyranoside)

Brown amorphous powder, Negative FAB-MS *m/z*: 477 [M-H]^−^, 1H-NMR, and 13C-NMR data [21].

#### 2.1.4. HIR (1,7-bis-(3,4-Dihydroxyphenyl)-4-Heptene-3-One)

Brown oil, Negative EI-MS *m/z*: 328 [M]^+^, 1H-NMR, and 13C-NMR data [21].

### 2.2. Experimental Model

#### 2.2.1. Induction of AD-Like Skin Lesions in NC/Nga Mice

Thirty-five female NC/Nga mice aged 3 weeks were purchased from SLC Tokyo (Tokyo) and maintained under conventional conditions. *Dermatophagoides farina* (House dust) containing cream was used to induce AD-like skin lesions. The back fur of ether-anaesthetized animals was shaved off using a hair clipper 1 week before sensitization. Induction was performed 14 days after sensitization. *Dermatophagoides farina* (House dust) containing cream was applied to backs twice a week from 3 to 17 weeks [22, 23].

#### 2.2.2. Treatment and Severity Scores

Phosphate Buffered Saline (PBS), and 0.1% HIR and 1% HIR liquid solutions were injected intraperitoneally, twice a week, and base cream and 1% HIR topical cream were applied to exposed back skin daily for 4 weeks.

Severity of dermatitis was assessed macroscopically in a blinded fashion weekly using the following scoring procedure. Total clinical skin severity scores were defined as the sum of individual scores for the following five signs and symptoms: itching, erythema, excoriation, scaling, and dryness. Each of these items was allocated scores of 0–3, where 0 = no symptoms, 1 = mild, 2 = moderate, and 3 = severe, as described previously [23–25].

#### 2.2.3. Measurement of Total IgE Level in Plasma

Blood was collected from the retro-orbital plexus using heparinized glass capillary tubes before and after treatment. Plasma samples were obtained by centrifuging at 12,000 rpm for 10 min and stored at −80°C until required for assay. Total plasma IgE levels were determined by enzyme-linked immunosorbent assay (ELISA) using a method involving the capture and detection of monoclonal antibody pairs, as suggested by BD Pharmingen (San Diego, CA).

#### 2.2.4. Eosinophil Count in Blood

Blood samples were collected before and after treatment. Whole blood cell counts were conducted on 30 ul samples diluted sixfold with 150 ul of saline. Differential counts were determined by counting under a microscope using Wring-Giemsa stained blood smears. Eosinophil counts were calculated from differential counts.

#### 2.2.5. Cytokine Assays in Splenocytes and Serum

Total splenocytes were prepared and cells were plated at 1 × 10^6^ cell/ml. Red blood cells were lysed using RBC lysis buffer (Sigma, St. Louis). Lysates were centrifuged at 13,000 rpm for 15 min at 4°C. Supernatants were removed and cells were seeded at 1 × 10^6^ cell/ml and cultured in 24 well plates. Supernatants containing cultured lymphocytes were harvested. Blood samples were collected from aortas. Serum samples were obtained by centrifuging at 13,000 rpm for 10 min and stored at −80°C until required for assay. The supernatants of cultured splenocytes and serum were analyzed for cytokine levels by ELISA.

#### 2.2.6. Real-Time RT-PCR

Tissues from AD-like skin lesions were suspended in TRIzolReagent (Invitrogen Life Technologies, Carlsbad, CA). Total RNA was extracted using TRIzol reagent according to the protocol provided by the manufacturer (Invitrogen Life Technologies, Carlsbad, CA). Equal amounts (2 ug) of total RNA were obtained from each sample and reverse transcribed into cDNA with oligo(dT)_15_ primers using M-MLV reverse transcriptase (Promega, Madison, WI) at 42°C for 1 h. Reaction mixtures were amplified using a SYBRGreen Supermix (Bio-Rad, Hercules, CA) in the presence of 1ul of the sense and of the antisense primers of COX-2 and iNOS, and with primers for GAPDH using a Cycler Real-Time PCR Detection system using the following thermal cycling program, 30 times at 94°C for 30 s, at 60°C for 30 s, and at 72°C for 40 s. The sequences of the primers used were as follows: mouse iNOS, 5′-ctgcctcttccaggtgt-3′ and 5′-gagggactttctgaatc-3′; mouse COX-2, 5′-cccacccatggcaaattccatggca-3′ and 5′-ggtgctgcttgttaggaggtcaagtaa-3′; and mouse GAPDH 5′-ccacccatggcaaattccatggca-3′ and 5′-ccctgttgctgtagccgtat-3′. The experiments were performed in triplicate and repeated at least three times.

#### 2.2.7. Western Blot Analysis

Tissues from AD-like skin lesions were suspended in ProprepReagent (iNtRON, Kyunggi, SN). Proteins were extracted using reagent according to the protocol provided by the manufacturer (iNtRON, Kyunggi, SN). After electrophoresis, proteins were transferred onto nitrocellulose membranes, which were blocked with 5% skim milk in Tris-buffered saline solution containing 0.1% Tween-20. Immunoblotting with primary antibody, anti-iNOS, and anti-COX-2 was followed by mouse anti-rabbit peroxidase conjugated antibody (Chemicon). Blots were developed using ECL solution.

#### 2.2.8. Statistical Analysis

The results are expressed as mean ± SD. The paired *t*-test was used to compare two groups and one-way ANOVA and Dunnett's *t*-test were used for multiple comparisons. The analysis was conducted using GraphPad Prism software. Statistical significance was accepted for *P*  value  of < .05.

## 3. Results

### 3.1. HIR Suppressed the Development of AD-Like Skin Lesions in NC/Nga Mice

**Figure 2:**
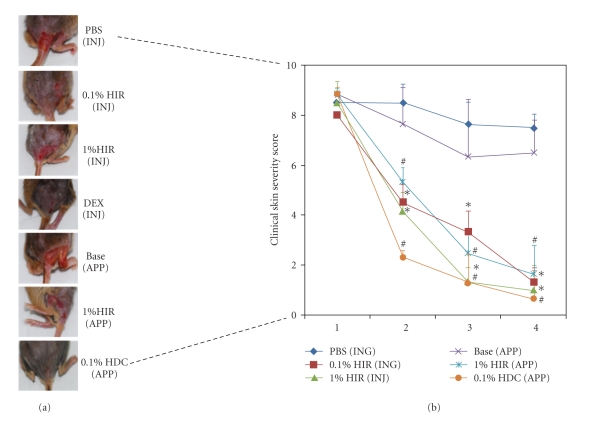
Progression of clinical dermatitis score for different intervention groups during the 4 weeks treatment period. Clinical findings were scored at 1-week intervals. (a) Representative clinical feature of NC/Nga mice skin. (b) HIR treatment significantly lowered total clinical severity scores. Data shown are mean ± S.D. of changes in total clinical dermatitis scores for face, ears, nose, and back. HIR: Hirsutenone, DEX: dexamethasone, Base: Baseline, HDC: hydrocortisone cream, INJ: injection, APP: application. *P < .05 compared with the PBS group. ^#^ *P* < .05 compared with the Base group. *n* = 5/group.

NC/Nga mice has been shown to develop AD-like skin lesions after the repeated application *Dermatophagoides farina* (House dust) containing cream. In accordance with this previous finding, clinical severity in the control PBS-injected and Base cream-treated NC/Nga mice increased gradually with number of challenges and reached a maximum on day 19 after sensitization. Most of the mice expressed AD-like skin lesions characterized by itching, erythema, excoriation, scaling, and dryness. The HIR, DEX, and 0.1% HDC exhibited lesion suppression, and clinical skin severity scores were significantly different in the control group and HIR groups (*P < .05 compared with the PBS group. ^#^ *P* < .05 compared with the base group) (Figure 2).

### 3.2. HIR Significantly Reduced Eosinophil Counts in Blood

**Figure 3:**
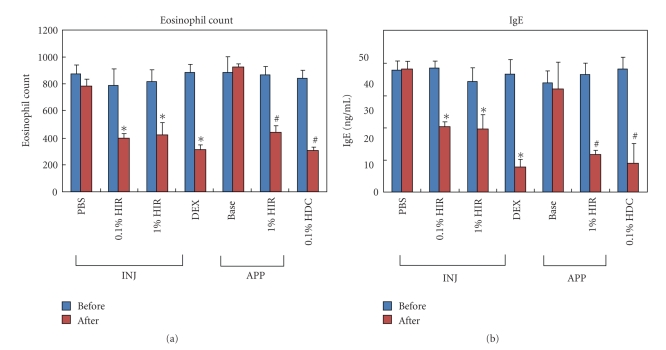
Changes in eosinophil counts and IgE levels before-to-after HIR treatment. Numbers of eosinophils reduced after HIR treatment. HIR significantly reduced plasma IgE levels. Data are shown as mean ± S.D. HIR = Hirsutenone, DEX = dexamethasone, Base = Baseline, HDC = hydrocortisone cream, INJ = injection, APP = application. *P < .05 compared with the PBS group. ^#^ *P* < .05 compared with the Base group. *n* = 5/group.

To investigate the effect of HIR treatment, we counted eosinophils in blood. The presence of eosinophils in the inflammatory infiltrate of AD has long been established, and thus blood was collected from the retro-orbital plexus. The PBS and Base groups showed increases in eosinophil counts before treatment. On the other hand, HIR significantly decreased eosinophil count after treatment commencement (*P < .05 compared with the PBS group. ^#^ *P* < .05 compared with the base group) (Figure 3(a)).

### 3.3. HIR Modulated Total IgE Levels

We performed ELISA to examine the effect of HIR on IgE production in the plasma induced by mite cream in NC/Nga mice. As shown in Figure 3(b), plasma IgE levels in the HIR group were significantly reduced compared to those of the control group. These results indicate that HIR had a significant suppressive effect on IgE production in plasma in the NC/Nga mice (*P < .05 compared with the PBS group. ^#^ *P* < .05 compared with the base group) (Figure 3(b)).

### 3.4. HIR Inhibited the Elevation of Th2-Related Cytokines in Serum and Lyphocytes

**Figure 4:**
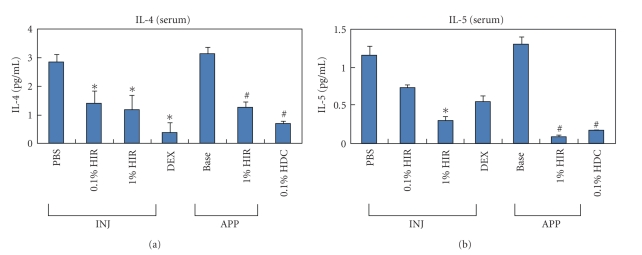
Changes in cytokine levels after HIR treatment. HIR significantly decreased the levels of IL-4 and IL-5 in the AD-like skin lesions of NC/Nga mice, but IL-13 levels were not significantly decreased. Analyses were repeated at least three times. Data are mean ± S.D. HIR: Hirsutenone, DEX: dexamethasone, Base: Baseline, HDC: hydrocortisone cream, INJ: injection, APP: application. *P < .05 compared with the PBS group. ^#^ *P* < .05 compared with the Base group. *n* = 5/group.

The effects of HIR on the regulatory cytokines related to AD, the cytokine levels for IL-4, IL-5, and IL-13 were quantified by ELISA. As shown in Figure 4, the levels of IL-4, IL-5, and IL-13 were significantly down regulated by HIR treatment, as compared to that of control group. These results indicate that HIR significantly reduced IL-4, IL-5, and IL-13 levels in the AD-like skin lesions (*P < .05 compared with the PBS group. ^#^ *P* < .05 compared with the base group) (Figure 4). 

### 3.5. HIR Treatment Significantly Down Regulated the mRNA and Protein Expressions of Inflammatory Factors

**Figure 5:**
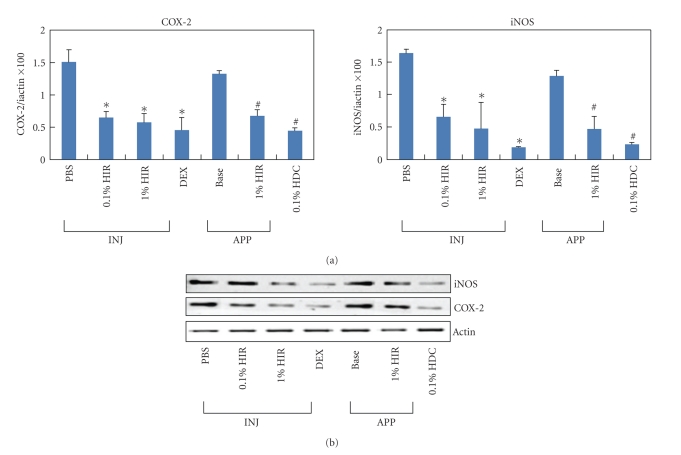
(a) COX-2 and iNOS expressions in AD-like lesions. (A) *Real-time* RT-PCR. (b) Western blots. Columns show means and standard deviations. The analyses were repeated at least three times. Data are mean ± S.D. HIR: Hirsutenone, DEX: dexamethasone, Base: Baseline, HDC: hydrocortisone cream, INJ: injection, APP: application. *P < .05 compared with the PBS group. ^#^ *P* < .05 compared with the Base group. *n* = 5/group.

To investigate the effect of HIR treatment on the inflammatory factors related to AD further, the mRNA and protein levels for COX-2, iNOS were quantified by *real-time *PCR. Both COX-2 and iNOS expressions were significantly lower in the HIR group, as compared to that of the control group. These results had shown that HIR reduced the expressions of COX-2 and iNOS in the AD-like skin lesions (*P < .05 compared with the PBS group. ^#^ *P* < .05 compared with the base group) (Figure 5). 

## 4. Discussion

In this study, we analyzed the therapeutic effect of HIR by applying it topically and injecting to NC/Nga mice with *Dermatophagoides farina* (House dust) containing cream-induced skin lesions. Although the therapeutic effects of HIR were less than those of HDC, which was used as an active control, topical application, and intraperitoneal injection treatment with HIR significantly reduced AD-like skin lesions and clinical signs provoked by *Dermatophagoides farina*, such as itching, erythema, excoriation, scaling, and dryness. Furthermore, topical application and intraperitoneal injection of HIR markedly reduced epidermal hyperplasia, eosinophil count, and IgE levels were of AD-like skin lesions. And Th2 cytokine levels and inflammatory factor levels reduced after HIR treatment.

 The presence of eosinophils in the inflammatory infiltrates of AD has been well established [26]. Previous studies on atopic dermatitis suggest that skin lesions accompanied by topical eosinophilia and systemic IgE elevation are associated with Th2 cytokines (IL-4, IL-5, and IL-13). Indeed, the elevated expressions of Th2 cytokines have been confirmed in both acute and chronic lesions of atopic dermatitis as compared with the uninvolved skins of patients with atopic dermatitis or those of normal subjects [27, 28]. It is known that IL-4, IL-5, and IL-13 are inflammatory cytokines that essential roles in the activation of T cells after antigenic stimulation, and that the skins of AD patients demonstrate Th2 cytokine overexpression, which has been reported to induce atopic responses, such as, itching, lichenification, and chronic inflammation [29]. 

 In our preliminary studies, we found that *Dermatophagoides farina*-treated NC/Nga mice showed progressive increases in total IgE during experimental period. Furthermore, in a separate experiment, total plasma IgE levels were found to be significantly elevated in *Dermatophagoides farina*-treated NC/Nga mice (data not shown). 

 Therefore, we devised an NC/Nga mice skin lesion model by repeatedly applying *Dermatophagoides farina* to investigate the clinical efficacy and the associated mechanism underlying the therapeutic effect HIR. 

Several reports have shown that natural immune modulators in herbal derivatives have therapeutic effects on AD and that HIR reduces the productions of Th1 and Th2 cytokines levels [13, 14, 30–32]. Accordingly, we investigated whether HIR modulates immune reaction in *Dermatophagoides farina*-treated NC/Nga mice. Our results show that HIR does suppress the development of AD-like skin lesions in NC/Nga mice. Furthermore, we found that the suppressive effects of HIR on these changes were paralleled by a decrease in total plasma IgE level, and that this might be due to the down-regulation of Th2 cytokines. It is also possible that the inhibition of mast cell infiltration and degranulation in skin by HIR reduces expressions of Th2 cytokines in serum and spleen. 

 Recently, innate immunity has been suggested to play an important role in the pathogenesis of AD [33, 34]. Nitric Oxide plays a role in vasodilation, neurotransmission, blood coagulation, and immune regulation. Furthermore, iNOS is a major producer of NO when it is activated by various cytokines or bacterial lipopolysaccaride (LPS). Therefore, one of the methods used to examine the anti-inflammatory activity of a compound is to measure its suppressive effects on the synthesis of iNOS [35]. COX-2 is markedly expressed in inflammation-related cells in response to stimulations by cytokines. 

In summary, we show here that HIR inhibits the development of AD-like skin lesions in NC/Nga mice induced by the repeated application of mite. The topical application and intraperitoneal injection of HIR could improve AD-like skin lesions in NC/Nga mice by inhibiting IgE, eosinophil count, or other Th2-related cytokines and inflammatory factors. Taken together, our finding suggested that the topical application and intraperitoneal injection of HIR may be a novel approach to the treatment of AD.

## Figures and Tables

**Figure 1:**
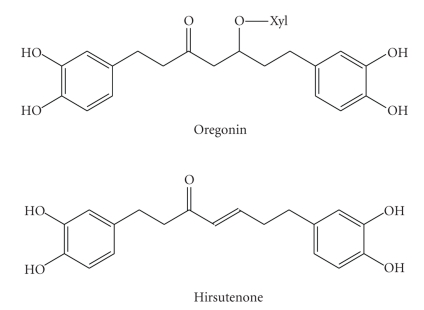
Chemical structures of oregonin and hirsutenone.

## References

[1] Simon D, Braathen LR, Simon H-U (2004). Eosinophils and atopic dermatitis. *Allergy*.

[2] Akdis CA, Akdis M, Trautmann A, Blaser K (2000). Immune regulation in atopic dermatitis. *Current Opinion in Immunology*.

[3] Thaçi D (2003). Long term management of childhood atopic dermatitis with calcineurin inhibitors. *Hautarzt*.

[4] Asakawa Y (1970). Chemical constituents of *Alnus firma* (BETULACEAE). I. Phenyl propane derivatives isolated from *Alnus firma*. *Bulletin of the Chemical Society of Japan*.

[5] Lee S-J (1966). *Korea Folk Medicine*.

[6] Lee Y-A, Jeong D-W, Kim K-H (2000). Antioxidant activity of diarylheptanoids from the leaves of *Alnus hirsute*. *Yakhak Hoeji*.

[7] Kuroyanagi M, Shimomae M, Nagashima Y (2005). New diarylheptanoids from *Alnus japonica* and their antioxidative activity. *Chemical and Pharmaceutical Bulletin*.

[8] Lee M-W, Kim N-Y, Park M-S (2000). Diarylheptanoids with in vitro inducible nitric oxide synthesis inhibitory activity from *Alnus hirsuta*. *Planta Medica*.

[9] Lee M-W, Kim J-H, Jeong D-W, Ahn K-H, Toh S-H, Surh Y-J (2000). Inhibition of cyclooxygenase-2 expression by diarylheptanoids from the bark of *Alnus hirsuta* var. *sibirica*. *Biological and Pharmaceutical Bulletin*.

[10] Kim H-J, Yeom S-H, Kim M-K, Shim J-G, Paek I-N, Lee M-W (2005). Nitric oxide and prostaglandin E2 synthesis inhibitory activities of diarylheptanoids from the barks of *Alnus japonica* steudel. *Archives of Pharmacal Research*.

[11] Han J-M, Woo SL, Kim J-R (2007). Effects of diarylheptanoids on the tumor necrosis factor-*α*-induced expression of adhesion molecules in human umbilical vein endothelial cells. *Journal of Agricultural and Food Chemistry*.

[12] Han J-M, Woo SL, Kim J-R (2008). Effect of 5-O-methylhirsutanonol on nuclear factor-*κ*B-dependent production of NO and expression of iNOS in lipopolysaccharide-induced RAW264.7 cells. *Journal of Agricultural and Food Chemistry*.

[13] Lee CS, Ko HH, Seo SJ (2009). Diarylheptanoid hirsutenone prevents tumor necrosis factor-*α*-stimulated production of inflammatory mediators in human keratinocytes through NF-*κ*B inhibition. *International Immunopharmacology*.

[14] Joo SS, Kim SG, Choi SE (2009). Suppression of T cell activation by hirsutenone, isolated from the bark of *Alnus japonica*, and its therapeutic advantages for atopic dermatitis. *European Journal of Pharmacology*.

[15] Uehara M, Kimura C (1993). Descendant family history of atopic dermatitis. *Acta Dermato-Venereologica*.

[16] Van Bever HP (1992). Recent advances in the pathogenesis of atopic dermatitis. *European Journal of Pediatrics*.

[17] Kishimoto T, Hirano T (1988). Molecular regulation of B lymphocyte response. *Annual Review of Immunology*.

[18] Reinhold U, Pawelec G, Wehrmann W, Herold M, Wernet P, Kreysel HW (1988). Immunoglobulin E and immunoglobulin G subclass distribution in vivo and relationship to in vitro generation of interferon-gamma and neopterin in patients with severe atopic dermatitis. *International Archives of Allergy and Applied Immunology*.

[19] Ried JL, Collmer A (1986). Comparison of pectic enzymes produced by *Erwinia chrysanthemi*, *Erwinia carotovora* subsp. carotovora, and Erwinia carotovora subsp. atroseptica. *Applied and Environmental Microbiology*.

[20] Schols HA, Voragen AGJ, Visser J, Voragen AGJ (1996). Complex pectins: structure elucidation using enzymes. *Pectins and Pectinases*.

[21] Kim HJ, Kim KH, Yeom SH (2005). New diarylheptanoid from the barks of *Alnus japonica* steudel. *Chinese Chemical Letters*.

[22] Sasakawa T, Higashi Y, Sakuma S (2001). Atopic dermatitis-like skin lesions induced by topical application of mite antigens in NC/Nga mice. *International Archives of Allergy and Immunology*.

[23] Yamaguchi T, Maekawa T, Nishikawa Y (2001). Characterization of itch-associated responses of NC mice with mite-induced chronic dermatitis. *Journal of Dermatological Science*.

[24] Matsuda H, Watanabe N, Geba GP (1997). Development of atopic dermatitis-like skin lesion with IgE hyperproduction in NC/Nga mice. *International Immunology*.

[25] Yamamoto M, Haruna T, Yasui K (2007). A novel atopic dermatitis model induced by topical application with *Dermatophagoides farinae* extract in NC/Nga mice. *Allergology International*.

[26] Simon D, Braathen LR, Simon H-U (2004). Eosinophils and atopic dermatitis. *Allergy*.

[27] Hamid Q, Boguniewicz M, Leung DYM (1994). Differential in situ cytokine gene expression in acute versus chronic atopic dermatitis. *Journal of Clinical Investigation*.

[28] Ohmen JD, Hanifin JM, Nickoloff BJ (1995). Overexpression of IL-10 in atopic dermatitis: contrasting cytokine patterns with delayed-type hypersensitivity reactions. *Journal of Immunology*.

[29] Fiset P-O, Leung DYM, Hamid Q (2006). Immunopathology of atopic dermatitis. *Journal of Allergy and Clinical Immunology*.

[30] Kotani M, Matsumoto M, Fujita A (2000). Persimmon leaf extract and astragalin inhibit development of dermatitis and IgE elevation in NC/NGa mice. *Journal of Allergy and Clinical Immunology*.

[31] Oku H, Ishiguro K (2001). Antipruritic and antidermatitic effect of extract and compounds of *Impatiens balsamina* L. in Atopic dermatitis model NC mice. *Phytotherapy Research*.

[32] Ziment I, Tashkin DP (2000). Alternative medicine for allergy and asthma. *Journal of Allergy and Clinical Immunology*.

[33] Cookson W (2004). The immunogenetics of asthma and eczema: a new focus on the epithelium. *Nature Reviews Immunology*.

[34] Strachan DP (2000). Family site, infection and atopy: the first decade of the ’hygiene hypothesis’. *Thorax*.

[35] Yamamoto M, Haruna T, Yasui K (2007). A novel atopic dermatitis model induced by topical application with *Dermatophagoides farinae* extract in NC/Nga mice. *Allergology International*.

